# Advances in Immunotherapy for Adult Glioblastoma

**DOI:** 10.3390/cancers13143400

**Published:** 2021-07-07

**Authors:** Chirayu R. Chokshi, Benjamin A. Brakel, Nazanin Tatari, Neil Savage, Sabra K. Salim, Chitra Venugopal, Sheila K. Singh

**Affiliations:** 1 Department of Biochemistry and Biomedical Sciences, McMaster University, Hamilton, ON L8N 3Z5, Canada; chokshc@mcmaster.ca (C.R.C.); brakelb@mcmaster.ca (B.A.B.); tatarin@mcmaster.ca (N.T.); savagen@mcmaster.ca (N.S.); salims@mcmaster.ca (S.K.S.); 2 Department of Surgery, Faculty of Health Sciences, McMaster University, Hamilton, ON L8N 3Z5, Canada; venugop@mcmaster.ca

**Keywords:** glioblastoma, immunotherapy, vaccine, immune checkpoint inhibitors, chimeric antigen receptor (CAR) T cells

## Abstract

**Simple Summary:**

Therapy failure and disease recurrence are hallmarks of glioblastoma (GBM), the most common and lethal tumor in adults that originates in the brain. Despite aggressive standards of care, tumor recurrence is inevitable with no standardized second-line therapy. Recent clinical studies evaluating therapies that augment the anti-tumor immune response (i.e., immunotherapies) have yielded promising results in subsets of GBM patients. Here, we summarize clinical studies in the past decade that evaluate vaccines, immune checkpoint inhibitors and chimeric antigen receptor (CAR) T cells for treatment of GBM. Although immunotherapies have yet to return widespread efficacy for the majority of GBM patients, critical insights from completed and ongoing clinical trials are informing development of the next generation of therapies, with the goal to alleviate disease burden and extend patient survival.

**Abstract:**

Despite aggressive multimodal therapy, glioblastoma (GBM) remains the most common malignant primary brain tumor in adults. With the advent of therapies that revitalize the anti-tumor immune response, several immunotherapeutic modalities have been developed for treatment of GBM. In this review, we summarize recent clinical and preclinical efforts to evaluate vaccination strategies, immune checkpoint inhibitors (ICIs) and chimeric antigen receptor (CAR) T cells. Although these modalities have shown long-term tumor regression in subsets of treated patients, the underlying biology that may predict efficacy and inform therapy development is being actively investigated. Common to all therapeutic modalities are fundamental mechanisms of therapy evasion by tumor cells, including immense intratumoral heterogeneity, suppression of the tumor immune microenvironment and low mutational burden. These insights have led efforts to design rational combinatorial therapies that can reignite the anti-tumor immune response, effectively and specifically target tumor cells and reliably decrease tumor burden for GBM patients.

## 1. Introduction

Glioblastoma (GBM) remains the most aggressive and prevalent malignant primary brain tumor in adults [1]. Unchanged since 2005, patients undergo standard of care (SoC) that consists of gross total resection to remove the tumor bulk, followed by radiation therapy (RT) with concurrent and adjuvant chemotherapy with temozolomide (TMZ) [2,3]. Despite these aggressive therapeutic efforts, tumor relapse is inevitable, and patients face a median overall survival of 14.6 months and a 5-year survival rate of 5.5–6.8% [1,2,4]. A major contributor to treatment failure is intra-tumoral heterogeneity that gives rise to tumor cell populations distinct at the genomic, transcriptomic, proteomic and functional levels [5,6,7,8,9]. In addition to SoC, two therapeutics have received approval from the Food and Drug Administration, including (1) an anti-vascular endothelial growth factor (VEGF) monoclonal antibody bevacizumab, and (2) tumor-treating fields that target proliferating tumor cells. However, these therapies have yet to be incorporated into SoC for GBM patients.

**Figure 1:**
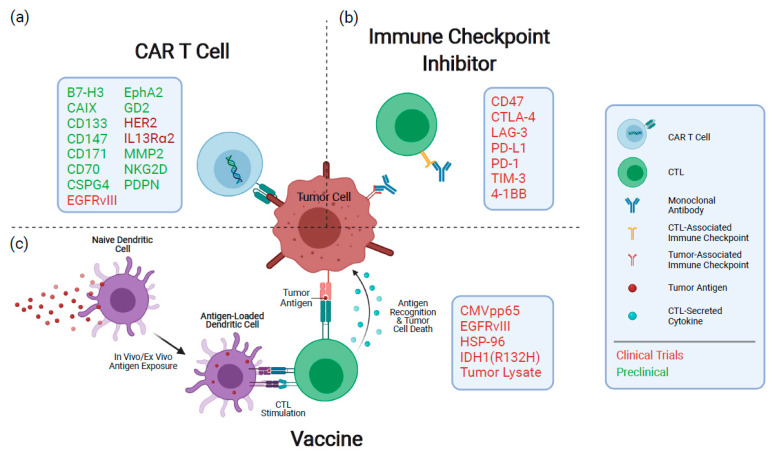
Overview of current immunotherapeutic modalities being investigated to treat GBM. (**a**) CAR T cells recognize antigens through a genetically engineered extracellular receptor which triggers intracellular T cell activation and degranulation upon antigen binding. (**b**) Inhibitors of immune checkpoint proteins prevent their attenuation of immune responses upon activation and exhaustion. (**c**) Vaccines expose antigen-presenting cells to tumoral antigens, stimulating a target-specific immune response. Boxes indicate therapeutic targets or mediators being pursued for each modality. CAR: chimeric antigen receptor; CTL: cytotoxic T lymphocyte.

Emerging therapeutics for GBM have shifted towards reconfiguring the patient’s immune system to generate an anti-tumor response. Here, we will summarize clinical findings and highlight promising preclinical studies of three major immunotherapeutic modalities designed to treat GBM, including vaccines, antibodies and chimeric antigen receptor (CAR) T cells (Figure 1). For a recent review of advances in oncolytic virotherapy for gliomas, refer to Rius-Rocabert et al. [10]. Given that resistance to SoC and disease relapse are inevitable for GBM patients, preclinical and clinical advancement of immunotherapeutic modalities, combined with recent insights into the tumor immune microenvironment, are poised to improve clinical outcomes for this patient population.

## 2. Vaccines

Cancer vaccines function by exposing tumor-associated antigens to antigen-presenting cells (APCs), which activate immune effector cells to achieve an anti-cancer immune response. Several promising vaccines targeting both single and multiple antigens have shown varying degrees of clinical response (Table 1); however, vaccines for GBM have yet to translate to SoC. While GBM-specific targets are sparse, several have been identified that are expressed exclusively or enriched in tumor cells. Perhaps the most explored to date, epidermal growth factor receptor variant III (EGFRvIII) is a mutant version of the EGFR receptor specificically-expressed in GBM and has been targeted extensively through a variety of immunotherapeutic efforts, including vaccination. Similarly, the cytomegalovirus (CMV) tegument phosphoprotein 65 (pp65) and IDH1 (R132H)-mutant peptides are frequently and specifically expressed in GBM, in contrast to healthy brain tissues [11,12]. Vaccination strategies targeting these proteins have shown efficacy in clinical trials and often elicit strong immune responses; however, no targets identified to date are expressed on all GBM cells, likely allowing clonally driven recurrence to evade such treatments. In contrast, multi-targeted vaccines initiating an immune response to multiple tumor-associated antigens better address intratumoral heterogeneity; however, these treatments have shown limited clinical success.

Antigen presentation and the following activation and regulation of effector cells is another important process in achieving an effective immune response, which involves several proteins such as those mediating suppression of T cells, macrophages and other tumor-infiltrating lymphocytes. Current efforts acting on this front, such as antibodies against these suppressors, have shown preclinical promise but have fallen short in clinical trials. Additionally, success seems to vary greatly upon the combination of these inhibitors, underlining the importance of understanding and enhancing synergistic interactions among treatments.

### 2.1. Single-Target Vaccines

Several vaccines have been developed for GBM targeting a single, tumor-specific antigen. One such vaccine is rindopepimut, a peptide vaccine targeting EGFRvIII which has been identified as a tumor-specific mutant expressed in roughly one-third of GBM specimens [27]. This protein enhances GBM tumorigenicity [28,29] and is highly immunogenic [30], altogether providing a promising target for immunotherapy. Early preclinical studies have confirmed its immunogenicity and shown it to be effective in mice [31]; however, the protein’s heterogeneous and unstable expression leaves room for EGFRvIII-negative tumor cells to drive therapy resistance and recurrence. A series of phase II rindopepimut trials, named “ACTIVATE, ACT II and ACT III,” have shown promise (NCT00643097, NCT00458601), achieving median survival times between 22 and 26 months [13,14,15]. To validate these findings, a large phase III, trial termed “ACT IV”, was completed with 371 patients (NCT01480479); however, no survival benefit was seen among vaccinated patients compared to controls, with median survivals of 20.1 and 20 months, respectively [16]. Interestingly, patients with significant residual disease received a greater benefit from the vaccine, perhaps due to a greater antigen load. Patients in the trial also showed strong humoral immune responses, suggesting resistance to the therapy was enabled at least in part by the heterogeneity of EGFRvIII expression. Indeed, those who underwent post-treatment biopsies of the recurrent tumor in both control and vaccinated groups showed loss of EGFRvIII expression in a majority of patients. This loss of expression highlights the limitations of single-target therapies in a heterogeneous tumor and underlines the importance combinatorial therapies will have in the future [32]. Additionally, the improved survival of the placebo group compared to historical controls was surprising, and future trials should account for this difference or change in control performance over time.

The complex interplay among therapies and the immune response must also be considered. For instance, rindopepimut was given along with TMZ, which induces lymphopenia [33]. While an accompanying increase in regulatory T cells suggests this may hinder the response to rindopepimut, previous findings have shown it can enhance it [14]. An additional study on rindopepimut was completed in 72 recurrent GBM patients in a phase II trial, termed “ReACT” (NCT01498328), combining the vaccine with bevacizumab, a monoclonal antibody against VEGF that has been shown to enhance immune responses [34]. The trial showed improvement upon the ACT IV trial, with 20% of treated patients surviving for 24 months compared to 3% for control-treated patients, in addition to a potential for rindopepimut to be combined with bevacizumab [17].

Another promising vaccination effort is the CMV dendritic cell (DC) vaccine. While rare in the healthy brain, viral proteins and nucleic acids of CMV are present in approximately 90% of GBM tumors [11]. The implications of CMV in tumor initiation and therapy resistance are not well understood; however, these viral antigens pose a potential immunotherapeutic target specific to cancerous cells. Of these antigens, CMV pp65 is highly expressed in glioma tumors and is the main target of current CMV vaccination strategies, as it elicits a strong cytotoxic T lymphocyte response following infection [35]. The CMV pp65 DC vaccine consists of autologous DCs pulsed with pp65 RNA fused in frame with the human Lysosomal Associated Membrane Protein (hLAMP) gene shown to enhance antigen processing [36]. A series of large phase II trials were recently completed with the vaccine in patients with newly diagnosed GBM following SoC treatment.

The initial “ATTAC” trial (NCT00639639) and subsequent “ATTAC-GM” trial (NCT00639639) both showed long-term survival in approximately one-third of patients. The initial trial also revealed that pre-conditioning with tetanus-diphtheria (Td) toxoid significantly increased DC migration to the lymph nodes, which correlated with increased survival, leading to half of the pre-conditioned patients remaining progression-free >36.6 months post diagnosis [18]. The second trial instead administered dose-intensified TMZ (DI-TMZ) with the vaccination, as DI-TMZ-induced lymphopenia has previously been shown to enhance both humoral and cellular immune responses [37]. While DI-TMZ increased immunosuppressive regulatory T cells, the group had a median survival of 41.1 months, greatly exceeding matched historical controls [19]. Excitingly, four patients remained progression-free at 59–64 months post-diagnosis, and overall, the trial showed the vaccine to be effective at targeting GBM based on the presence of CMV pp65. A subsequent phase II trial termed “ELEVATE” is ongoing to validate the benefit of Td toxoid pre-conditioning on DC migration and to evaluate synergy among vaccination, Td toxoid pre-conditioning and the anti-tumor antibody basiliximab (NCT02366728). To date, the trial has confirmed increased migration of DCs to the lymph nodes following pre-conditioning; however, analysis of other aims is not yet complete [20].

Vaccines have also been developed targeting the IDH1 subtype of gliomas, consisting of the IDH1 (R132H)-mutated peptide, which is present in <15% of GBM patients [12]. The vaccine was previously found to be effective in a mouse model transgenic for human MHC class I and II with IDH1 (R132H), showing MHC class II presentation of the epitope and mutation-specific T cell and antibody responses [38]. A phase I clinical trial termed “NOA-16” (NCT02454634) was recently completed for the vaccine delivered concurrently with topical imiquimod, a myeloid-activating TLR7 agonist. Results of the trial were extremely promising, with 93% of grade III-IV glioma patients showing a vaccine-specific immune response and 84% surviving >3 years [21]. A second phase II trial called “RESIST” is underway, adjuvating the vaccination with granulocyte-macrophage colony-stimulating factor (GM-CSF) in combination with TMZ and Td toxoid (NCT02193347).

### 2.2. Multi-Target Vaccines

To treat a heterogeneous disease such as GBM, targeting a single antigen can lead to clonal evolution and drive resistance. One way of overcoming this is by targeting multiple antigens concurrently. Interestingly, the greatest progress in therapeutic development has thus far been observed for single antigen-targeting vaccines, likely due to tumor-specific expression of these antigens. Regardless, the importance of targeting the molecular heterogeneity of GBM tumors is well established, and several multi-targeted GBM vaccines have shown promising results, such as personalized neoantigen-based vaccination strategies [39]. One such multi-targeted vaccine is DCVax-L, a personalized approach to peptide vaccination that uses autologous, or patient-derived, DCs pulsed with resected tumor lysate to target a variety of tumor antigens. In rat models, the vaccine was found to significantly increase survival and T cell infiltration [40], leading to several clinical trials. In a phase III trial (NCT00045968), a subset of patients (*n =* 232) were vaccinated and given concurrent TMZ, while all patients (*n =* 331) were given the vaccine upon tumor recurrence. The overall study population had a median survival of 23.1 months, with a large group (*n =* 100) having a particularly long median survival of 40.5 months unexplained by any prognostic factors, suggesting clinical efficacy related to vaccination [22]. A trial is now ongoing in patients who were previously ineligible due to post-chemoradiotherapy progression or insufficient vaccine production (NCT02146066). As an alternative approach to pulsing DCs with tumor lysate, DCs pulsed with a synthetic cocktail of tumor-associated antigens have shown promising preliminary results, with 5 of 16 vaccine-treated GBM patients surviving 6 years post-diagnosis [41,42].

Vaccines relying on heat shock proteins (HSP) are also being explored for GBM treatment. There have been several trials investigating HSP vaccines for glioma, which consist of HSPs and tumor-associated peptides. These vaccines primarily rely on tumor-derived HSP glycoprotein 96 (gp96), which binds tumor antigens forming the HSP protein complex-96 (HSPPC-96). This complex mediates presentation of antigens in antigen-presenting cells and can bind different peptides for a multi-targeted approach. An initial trial of a multi-peptide HSPPC-96 vaccine with TMZ (NCT00293423) confirmed strong peripheral and local immune responses specific to HSPPC-96-bound antigens in 11 of 12 treated patients [23]. These responders had a median survival of 11.8 months post-vaccination and surgery compared to 4 months for the single non-responding patient, and in the phase II portion of this trial, patients showed a median survival of 10.7 months, significantly exceeding controls [24]. Additionally, patients with pre-vaccination lymphopenia had decreased survival compared to those with higher lymphocyte counts, likely due to worsened immune function and thus decreased responses. Addressing this question and further validating effectiveness of this vaccine, another trial (NCT02122822) revealed those with strong tumor-specific immune responses indeed had longer median survival than those with weak responses (>40.5 months and 14.6 months, respectively), with the overall patient population reaching a median survival of 31.4 months and again exceeding controls [25].

Another phase II trial was recently completed with the HSPPC-96 vaccine and TMZ following SoC (NCT00905060), achieving a median survival of 23.8 months, further validating efficacy of this vaccine [26]. Interestingly, this trial found expression of the T cell-suppressing immune checkpoint PD-L1 in myeloid cells to be indicative of survival, with high expression leading to shorter survival as compared to patients with lower PD-L1 expression (18 months and 44.7 months, respectively). While a promising lead, no HSPPC-96 vaccines have been combined with anti-PD-L1 therapies to date. However, a trial is currently investigating the vaccine when combined with standard TMZ, radiotherapy and the antibody pembrolizumab targeting the PD-L1 receptor, which is ongoing (NCT03018288).

## 3. Antibodies Modulating the Tumor Immune Microenvironment

A complex system of stimulatory and inhibitory regulators functions to maintain immune homeostasis. An important part of this system is immune checkpoints, which regulate activation to avoid autoimmunity. Upon activation or exhaustion, several immune cells upregulate these inhibitory checkpoints, thus limiting the immune response. Cancer cells express immune checkpoint proteins as well, allowing them to suppress the anti-cancer immune response. As a result, antibodies against these checkpoints, known as immune checkpoint inhibitors (ICI), have shown success in several cancers such as melanoma and non-small-cell lung cancer [43], and several are being tested for GBM (Table 2). Of these antibodies, the greatest progress has been noted for ICIs blocking programmed cell death protein 1 (PD-1) and cytotoxic T lymphocyte antigen 4 (CTLA-4), which are expressed on T cells to inhibit T cell activation and killing of tumor cells [44,45].

### 3.1. Immune Checkpoint Inhibitors

PD-1 targeting antibodies pembrolizumab and nivolumab have been approved to treat various solid tumors [43]; however, widespread clinical efficacy in GBM has yet to be achieved. Combination of an anti-PD-1 antibody and radiotherapy has shown preclinical success in vivo [53], leading to the phase III CheckMate 143 trial of nivolumab (NCT02017717) comparing it to the approved VEGF-A inhibitor bevacizumab in recurrent GBM. The trial results showed a median survival of around 10 months for both groups and identical 12-month survival rates of 42% [46]. Additionally, preliminary safety data of an earlier cohort of patients revealed high toxicity of a previously considered anti-PD-1/anti-CTLA-4 combination arm [54], leading to the discontinuation of this dual ICI therapy. Nivolumab has also been explored in other combinations such as the phase III CheckMate 498 trial (NCT02617589) delivered with radiotherapy, as compared to SoC (TMZ and radiotherapy); however, the trial showed no survival advantage of nivolumab treatment with similar median survivals around 14 months for both groups. Another phase III trial, CheckMate 548 (NCT02667587), is combining nivolumab, radiotherapy and TMZ. While still ongoing, an announcement was made that the trial failed to meet its primary endpoints of overall survival and progression-free survival [47].

Pembrolizumab is another anti-PD-1 antibody currently in trial for treatment of gliomas. In a phase I trial of 24 recurrent, high-grade glioma patients treated with pembrolizumab, bevacizumab and hypofractionated stereotactic irradiation (NCT02313272), more than half the patients achieved significant responses, and median survival was 13.5 months [48]. However, another phase I trial of pembrolizumab with bevacizumab compared to pembrolizumab alone in recurrent GBM patients (NCT02337491) showed a median survival of 8.8 months and 10.3 months, respectively [49]. The reduced survival upon lack of radiotherapy emphasizes the potential synergy of radiotherapy with anti-PD-1 therapies.

The interplay among chemotherapy and ICIs can also impact therapeutic efficacy, with preclinical studies showing that the order, timing and administration of chemotherapy relative to anti-PD-1 therapy drastically alter responsiveness of GBM tumors [55]. Additional efforts have been made to enhance the anti-tumor response, including neoadjuvant ICI administration prior to surgery, which has enhanced and prolonged the anti-tumor immune response and increased survival in other cancers [56,57]. A phase II trial using this approach with pembrolizumab in recurrent GBM patients showed increased survival with neoadjuvant and post-surgery adjuvant treatment, as compared to post-surgery adjuvant-only treatment (13.2 months and 6.3 months, respectively) [58]. Neoadjuvant administration also led to an upregulation of T cell- and interferon-γ-related gene expression and down-regulation of cell cycle-related genes. In a similar phase II trial (NCT02550249), neoadjuvant nivolumab was shown to enhance chemokine expression, T cell receptor (TCR) clonal diversity among tumor-infiltrating lymphocytes (TILs) and immune-cell infiltration in the tumor; however, median survival of treated patients was only 7.3 months [50]. Interestingly, two patients in the neoadjuvant cohort had complete surgical resection and remained disease-free for 33.3 and 28.5 months, which was not explainable by any recorded prognostic factors.

CTLA-4 (CD152) is another ICI that reduces CD28 co-stimulatory signaling by competitively binding to its natural ligands CD80 and CD86, suppressing T cell stimulation. Anti-CTLA-4 therapy has been approved for several cancers [43], extending survival of glioma-bearing mice [59], and in combination with anti-PD-1 therapy, shown eradication of tumors in a majority of mice [60]. Clinical trials have recently begun assessing anti-CTLA-4 therapies in treating gliomas (NCT02311920, NCT02829931), though no trials have been completed with glioma patients to date.

PD-L1, the ligand of PD-1 regularly expressed on APCs, is also expressed in cancer cells and mediates suppression of tumor-infiltrating T cells. Anti-PD-L1 antibodies have been approved in other cancers [43]; however, their efficacy in gliomas remains poor. An ongoing phase II trial is evaluating the anti-PD-L1 antibody durvalumab with radiotherapy and bevacizumab in GBM (NCT02336165), with preliminary results of the recurrent, bevacizumab-refractory cohort showing only 36% survival at 5.5 months [51].

Another phase I trial is looking at a different combination of ICIs, treating recurrent glioma patients with durvalumab and an anti-CTLA-4 antibody (NCT02794883); however, no updates have been given. Combinations of the anti-PD-L1 ICI avelumab are also being investigated, with ongoing phase II trials testing combinations with both hypofractionated radiation therapy (NCT02968940) and chemoradiotherapy (NCT03047473). Previous trials have found low expression of PD-L1 in GBM, with the CheckMate-143 trial finding only 10 of 37 patients with evaluable PD-L1 expression showing ≥10% [54]. This inherently limits any PD-L1 targeted therapies and may partially explain poor clinical outcomes thus far.

LAG-3 is another immune checkpoint receptor expressed on exhausted T cells that negatively regulates T cell responses. While anti-LAG-3 therapies have shown preclinical success [61], LAG-3 is expressed in a small percentage of tumor-infiltrating lymphocytes [62], thus limiting the potential impact of these therapies on stimulating the immune response. Regardless, a phase I trial evaluating the anti-LAG-3 antibody “BMS 986016” is underway, assessing its efficacy alone and in combination with the anti-PD-1 antibody nivolumab in recurrent GBM patients (NCT02658981). A recent update revealed a median survival of 8 months for the anti-LAG-3 group and 7 months for the anti-LAG-3, anti-PD-1 combination group. The trial also assessed an agonistic antibody targeting the 4-1BB (CD137) immune checkpoint protein. 4-1BB is a co-stimulatory receptor expressed by T cells upon activation, which augments activation signaling. The anti-4-1BB group had a promising median survival of 14 months [52]; however, while preclinical investigations support this therapy [63,64], further trials with anti-4-1BB antibodies are required.

TIM-3 is a receptor expressed on lymphocytes that can suppress the immune response by inducing T cell exhaustion, such that expression of TIM-3 in GBM has been linked with poor patient prognosis [65]. Anti-TIM-3 antibody therapy for GBM has shown success preclinically in combination with anti-PD-1 therapy and stereotactic radiosurgery (SRS). SRS drives the release of antigens from the tumor, enhancing the immune response, which is further stimulated by concurrent checkpoint inhibitors. While neither anti-TIM-3 nor SRS alone prolonged survival of GBM-bearing mice, combining the two increased median survival from 22 to 100 days, an effect similarly obtained using an anti-TIM-3 and anti-PD-1 combination [66]. When combining all three treatments, 100% of mice were alive 100 days post-engraftment, revealing great synergy and prompting a phase I trial of this combinational therapy, which is underway (NCT03961971).

### 3.2. Macrophage-Targeted Antibodies

Response to ICIs varies among tumor types and may depend on immune infiltrates such as TILs. Recently, mass cytometry and single-cell RNA sequencing of patient tumor specimens from various ICI-responding and non-responding cancers, such as GBM, revealed enrichment of CD73-high macrophages in GBM, which persist through anti-PD-1 treatment and limit ICI efficacy by inhibiting T cell infiltration [67]. Prevalence of these CD73-expressing macrophages correlated with a low response to ICIs, and genetic perturbation of CD73 in mice improved efficacy of anti-CTLA-4 and anti–PD-1 combination therapy, which correlated with greater T cell infiltration. These results show a promising and novel immunotherapeutic target to combine with existing ICIs.

CD47 is an enzyme that suppresses macrophage activation through binding the signal regulatory protein α (SIRPα). CD47 is overexpressed in many tumors [68], allowing cancer cells to avoid phagocytosis. Anti-CD47 antibodies have been developed to shift macrophages to an immunostimulatory phenotype, promoting an anti-tumor response [69] and effectively reducing growth of several tumors [70,71]. Preclinical studies of anti-CD47 therapies for glioma have shown that, while anti-CD47 therapy is sometimes effective at stimulating glioma cell phagocytosis [72], chemotherapy and radiotherapy are synergistic with treatment and may be required to enhance phagocytosis and extend survival in mice [73,74]. This enhanced phagocytosis also leads to increased antigen cross-presentation and T cell priming [74], and anti-CD47 therapies have shown synergy with autophagy inhibition [75,76], as well as other ICIs and tumor-specific antibodies [77]. The potential for synergistic co-therapies sophisticates treatment with anti-CD47 antibodies, and effective combinations should be compared prior to therapeutic development efforts.

## 4. Chimeric Antigen Receptor (CAR) T Cells

Chimeric antigen receptor (CAR) T cells represent an efficacious form of adoptive T cell therapy, in which peripheral T cells are genetically engineered to express a fusion receptor protein (i.e., CAR) that recognizes and targets a tumor-specific or -enriched antigen. Rapid and rational evolution of receptor design has transformed the first-generation CAR—composed of a ligand-binding domain, extracellular spacer, transmembrane domain and an intracellular signaling domain—that suffered from limited signaling strength to highly efficacious second- and third-generation CARs that incorporate one or more intracellular co-stimulatory domains, respectively, to initialize and sustain T cell signaling [78,79,80,81]. Irrespective of design principles, an antigen-bound CAR T cell activates a potent cytokine release and cytolytic degranulation response that kills antigen-expressing tumor cells and results in T cell proliferation [82]. CAR T cell therapy has been highly effective against hematological malignancies, achieving remission rates of up to 90% in patients with relapsed or refractory B cell malignancies with anti-CD19 CAR T cells [83]. However, widespread clinical responses of CAR T cells have yet to be seen for solid tumors, including GBM. Here, we summarize lessons learned from clinical evaluation of CAR T cell therapies in GBM patients, highlight promising preclinical candidates and discuss approaches to improving clinical efficacy.

Unlike hematological malignancies, CAR T cell therapy design and administration require unique considerations in the context of GBM, including factors such as intratumoral antigen heterogeneity, bypassing the blood–brain barrier (BBB) and exerting a potent anti-tumor response in a highly immunosuppressive microenvironment [84]. Two schools of thought have guided the delivery of CAR T cell therapy to the brain thus far, one which supports systemic intravenous administration, and the other prefers intracavitary or intraventricular dosing to bypass the BBB. Supported by reports of a dysregulated BBB in GBM patients [85,86], investigators evaluating CAR T cell therapies targeting EGFRvIII and HER2 preferred intravenous delivery of their modality [87,88]. Although no dose-limiting toxicities were observed for either modality when delivered intravenously, three grade 2–4 adverse events were possibly associated with HER2 CAR T cell therapy, including headache (*n =* 1) and seizure (*n =* 2). In contrast, intracavitary (or intratumoral) delivery of CAR T cells is not functionally restricted by the BBB. Using a reporter gene system, preliminary clinical evidence supports trafficking of intracerebrally administered anti-IL13Rα2 CAR T cells to the tumor region using [^18^F]FHBG PET-based imaging [89]. Intracavitary treatment of GBM patients with anti-IL13Rα2 CAR T cells resulted in no dose-limiting toxicities [90,91]. However, similar to intravenous delivery of anti-EGFRvIII CAR T cells, two grade 3 adverse events were associated with the treatment, including headache (*n =* 1) and a neurologic event (*n =* 1). Unfortunately, an empirical and clinical comparison among CAR T cell delivery routes has yet to be performed for GBM.

To varying extents, clinical studies have evaluated CAR T cells for GBM targeting interleukin-13 receptor subunit alpha-2 (IL13Rα2), human epidermal growth factor receptor 2 (HER2) and EGFRvIII (Table 3), with follow-up studies targeting IL13Rα2 and HER2 underway. In addition, investigators have initiated clinical studies to evaluate CAR T cells targeting matrix metallopeptidase 2 (MMP2) [92], B7 family member B7-H3 [93,94,95], CD147 and NKG2-D type II integral membrane protein (NKG2D) [96,97]. Here, we outline clinical advances in CAR T cell therapies for the treatment of GBM.

### 4.1. IL13Rα2-Specific CAR T Cells

IL13Rα2 is a monomeric high-affinity receptor for interleukin 13 (IL13) that is enriched in GBM specimens compared to normal brain tissue [100,101]. In fact, IL13Rα2 expression correlates moderately with the mesenchymal signature [100], a subtype of GBM associated with greater proliferation, tumorigenicity and resistance to conventional chemoradiotherapy as compared to other subtypes [102,103]. Supported by these findings, IL13Rα2 CAR T cells were designed using a mutated IL13-zetakine binding domain (IL13.E13K.R109K), engineered to provide greater specificity for IL13Rα2 over IL13Rα1/IL4Rα and attached to a CD28 co-stimulation and CD3ζ signaling domain [104]. These IL13-zetakine CAR T cells were specifically and potently activated in the presence of IL13Rα2-expressing glioma cells, whereas no appreciable effect was seen in the absence of IL13Rα2 expression. Strikingly, a single intracranial injection of IL13-zetakine CAR T cells into mice with orthotopic glioma xenografts led to a robust decrease in tumor burden and increased median overall survival from 35 to 40 days in control mice to 88 days in IL13-zetakine CAR T cell-treated mice. These promising preclinical results led to the first-in-human pilot safety and feasibility study of IL13-zetakine CAR T cells in three patients with relapsed GBM [90]. In the study, IL13-zetakine CAR T cells were administered via an implanted reservoir/catheter system and led to treatment-induced inflammation at the tumor site. Although this treatment was well tolerated and led to decreased expression of IL13Rα2, two grade 3 headaches and a grade 3 neurologic event were observed following CAR T cell administration. A mean survival of 11 months after relapse was noted for these three patients, with one patient surviving 14 months.

Following this study, the group engineered second-generation IL13-targeted CAR T cells with a 4-1BB (CD137) co-stimulation domain and a mutated IgG4-Fc linker to improve anti-tumor potency and increase T cell persistence, while improving the safety profile [91,105]. These reengineered IL13BBζ-CAR T cells were administered to a patient with highly aggressive recurrent GBM with multifocal leptomeningeal disease and high IL13Rα2 expression. Although intracavitary infusions of IL13BBζ-CAR T cells did not cause any grade 3 or higher toxic effects and inhibited disease progression locally, distal non-resected tumors and new tumors progressed. Prompted by distant disease progression, IL13BBζ-CAR T cells were delivered via intraventricular infusions and led to dramatic reductions of all tumors after the fifth infusion, with a 77–100% decrease in tumor burden, a systemic anti-tumor inflammatory response and an absence of systemic toxic effects, allowing the patient to return to normal life and work activities. Unfortunately, disease recurrence was observed after 7.5 months with tumor formation in new locations and decreased expression of IL13Rα2, elucidating a common antigen loss response to targeted therapies and advocating for rational combinational or adjuvant therapies. Recently, preclinical efforts to improve IL13Rα2-directed CAR T cell therapy have included the incorporation of an IL13Rα2-specific single-chain variable fragment (scFv) [106], complementary IL15 expression to enhance T cell effector function [107], characterization of the tumor immune microenvironment following CAR T cell therapy [108] and optimal selection of T cell subsets for sustained CAR activity [109].

### 4.2. EGFRvIII-Specific CAR T Cells

Expressed heterogeneously in ~30% of GBM specimens [110], investigators have engineered and evaluated EGFRvIII-targeted CAR T cells in two in-human trials. A phase I study of EGFRvIII-targeted CAR T cells, previously tested in orthotopic xenograft models of EGFRvIII+ glioma for efficacy and specificity to EGFRvIII over EGFR [111,112], was conducted in 10 patients with EGFRvIII+ recurrent GBM to evaluate safety and feasibility as the primary endpoints [87]. Although no subjects experienced dose-limiting toxicities, including systemic cytokine release syndrome, tumor regression was not observed in any patients based on magnetic resonance (MR) imaging. A median overall survival of ~8 months was noted after CAR T cell infusion, with one long-term survivor exhibiting stable disease for >18 months. Of 10 treated patients, 7 underwent tumor resection post-infusion, and analysis of tumor tissue indicated a decrease or ablation of EGFRvIII expression. A second phase I clinical trial leveraged a third-generation EGFRvIII-targeted CAR with 4-1BB and CD38 co-stimulation domains to conduct a dose-escalation study in 18 patients with EGFRvIII+ GBM [99]. No dose-limiting toxicities were observed with EGFRvIII-targeted CAR T cells until the highest dose of ≥10^10^, at which point a patient developed acute dyspnea and experienced oxygen desaturation, eventually succumbing to severe hypotension. Despite efforts to increase CAR T cell persistence and tumor localization, no objective responses were noted using MR imaging, with 16 of 17 remaining patients showing signs of disease progression <3 months after infusion and a median survival of 6.9 months post-treatment. Interestingly, a single patient remained alive up to 59 months post-CAR therapy, and an additional two patients survived >1 year. In addition to further preclinical studies on third-generation anti-EGFRvIII CAR T cells by multiple groups [113,114,115], recent studies have augmented their approach to increase efficacy and decrease toxicity, including an approach to combine anti-EGFRvIII CAR T cells with anti-EGFR bispecific T cell-engager (BiTE) antibodies to treat EGFR-positive/EGFRvIII-negative GBM [116]. There are bispecific antibodies, such as BiTEs, that are synthetic antibody structures that bind to two separate epitopes, with intentions such as bridging tumor-immune cell interactions or increasing antibody specificity. An in-depth review of bispecific antibodies, including BiTEs, was recently presented by Lim et al. [117]. Moreover, investigators recently developed multi-antigen prime-and-kill synNotch-CAR T cells that use a dual receptor circuit, the first of which detects EGFRvIII or a brain-specific myelin oligodendrocyte glycoprotein to induce expression of CARs against EphA2 and IL13Rα2 [118]. In comparison to constitutively active anti-EGFRvIII/EphA2/IL13Rα2 CAR T cells, synNotch-CAR T cells showed greater anti-tumor efficacy without off-tumor toxicity.

### 4.3. HER2-Specific CAR T Cells

The human epidermal growth factor receptor 2 (HER2), originally discovered as a tumor-associated antigen in breast cancer, is a transmembrane glycoprotein with an intracellular tyrosine kinase domain [88]. HER2 is a sparsely expressed antigen in GBM, detected in up to 17% of specimens and indicative of poor prognosis [119,120]. With promising preclinical results of a second-generation anti-HER2 CAR engineered with a CD28 co-stimulatory domain [88], a clinical trial was undertaken to treat 17 patients with HER2-positive GBM with virus-specific anti-HER2 CAR T cells [98]. Although no dose-limiting toxicity was observed and CAR T cell persistence was noted up to 12 months post-infusion, no significant survival benefit was noted for treated patients with a median overall survival of 11.1 months.

## 5. Discussion

Immunotherapy has yet to significantly improve clinical outcomes for GBM patients, and clinical studies have been disappointing thus far. Here, we detailed clinical and preclinical advances in immune checkpoint blockade, vaccination strategies and emerging CAR T cell therapies for the treatment of GBM (Figure 1). Among the major hurdles to clinical efficacy are immense intratumoral heterogeneity [6,7], parallel modes of immunosuppression by tumor cells [121,122,123] and low mutational burden in GBM [124]. With these factors in mind, investigators and clinicians are shifting their focus to combinatorial and personalized treatment strategies to achieve synergistic effects, reduce treatment resistance and overcome immunosuppression.

Given their effectiveness in other cancers such as melanoma [125], ongoing clinical studies are combining ICIs with conventional chemoradiotherapy and experimental therapeutics to increase efficacy. A rational advancement of ICI therapy is co-targeting multiple immune checkpoints, with clinical trials initiated to test the following combinations in GBM: anti-CTLA4 and/or anti-PD-1 with TMZ in newly diagnosed GBM (NCT02311920), anti-CTLA-4 and anti-PD-L1 in recurrent GBM (NCT02794883), anti-LAG-3 and anti-PD-1 in recurrent GBM (NCT02658981), anti-IDO with anti-CTLA4 or anti-PD-1 in GBM (NCT02327078). In addition, hypofractionated stereotactic radiotherapy (NCT0289931, NCT02313272 and NCT02530502) and MRI-guided laser ablation (NCT02311582) are also being combined with ICI. As reviewed by Rius-Rocabert, Garcia-Romero, Garcia, Ayuso-Sacido and Nistal-Villan [10], oncolytic viruses are another form of immunotherapy that preferentially infect tumor cells, thereby activating the innate immune system and increasing T cell trafficking to the tumor bed. Based on promising preclinical data [126,127,128], clinical studies are evaluating a combination of adenovirus-based therapy DNX-2401 with anti-PD-1 blockade for recurrent GBM (NCT02798406). Furthermore, a preclinical study has confirmed the usefulness of an anti-PD-1 antibody at augmenting DC vaccination in glioma-bearing mice, showing a significant improvement in survival attributed to the strong T cell response enabled by ICI treatment [129]. Given that genetically engineered CAR T cells are exposed to the same immunosuppressive microenvironment as endogenous tumor-infiltrating lymphocytes, ICIs are being combined with CAR T cells to augment their performance. A phase I clinical trial is evaluating anti-IL13Rα2 CAR T cells as a single modality and in combination with ICIs Nivolumab and Ipilimumab (NCT04003649). Synergy among ICIs and other immunotherapeutic modalities will likely play a key role in advancing future therapies through addressing the immunosuppressive nature of the tumor.

Although CAR T cell therapy is a newer adaptation for GBM treatment, advancements to increase its clinical utility are rapidly progressing. Currently, 12 clinical trials are recruiting GBM patients to evaluate CAR T cell therapy against B7 family member B7-H3 (NCT04385173, NCT04077866), CD147, HER2 (NCT03389230), IL13Rα2 (NCT04003649, NCT04661384, NCT02208362), matrix metallopeptidase 2 (MMP2; NCT04214392) and NKG2D (NCT04717999). Furthermore, a recent clinical letter outlined the administration of B7-H3 CAR T cells to a 56-year-old woman with recurrent GBM, highlighting a potent but short-term anti-tumor response in situ, absent of grade 3 or higher toxicities associated with CAR T cell infusion [94]. Unfortunately, target antigen heterogeneity was predicted as the reason for treatment failure, as noted previously for CAR T cell therapy targeting EGFRvIII and IL13Rα2 [87,91]. Additionally, novel therapeutic targets for CAR T cell therapy are quickly emerging, including antigens such as the disialoganglioside GD2 [130], CD70 [131,132], CD133 [133], carbonic anhydrase IX (CAIX) [134], EphA2 [135,136], podoplanin (PDPN) [137], chondroitin sulfate proteoglycan 4 (CSPG4) [138,139] and adhesion molecule L1-CAM (CD171) [140]. Of these antigens, EphA2 is part of the EphR receptor tyrosine kinase family that coordinates positioning and patterning during early development [141]. Given that EphA2 is overexpressed in GBM specimens, especially in post-therapy GBM stem-like cells [142], anti-EphA2 CAR T cells [135,136] may be suited to target GBM at tumor recurrence. While current trials are focused on targeting single tumor-associated antigens, this increased repertoire of targets will allow multiple antigens to be targeted concurrently to overcome intertumoral heterogeneity. This approach has yielded fruitful results in preclinical glioma models, as shown by the development of tandem CAR T cells that bind HER2 and IL13Rα2 [143], as well as trivalent CAR T cells targeting HER2, IL13Rα2 and EphA2 [144]. In fact, these trivalent CAR T cells were able to eradicate nearly 100% of tumor cells from multiple GBM samples.

In addition to tumor-targeted CAR T cells and ICIs, modalities acting on other parts of the tumor immune microenvironment may play a vital role in achieving effective anti-tumor responses in a clinical setting. We summarized macrophage-targeted antibodies in Section 3.2 of this article. Another approach stems from a recent study that found natural killer cell function to be altered upon tumor infiltration, showing impairing lytic function as a possible mechanism of tumor immune evasion [145]. Strategies aimed at restoring natural killer cell activity against GBM are being investigated and have shown preclinical promise.

## 6. Conclusions

Emerging trends towards rational combinatorial therapies are likely to include a systemic reignition of the tumor immune microenvironment. The continued discovery of novel tumor-associated and tumor-specific antigens, paired with the improvement of therapeutic modalities to increase efficacy and reduce toxicity, are necessary for the clinical efficacy of immunotherapies. Overall, a combinatorial therapy delivered at various stages throughout SoC may reliably improve clinical outcomes in GBM patients.

## Figures and Tables

**Table 1:** Summary of clinical trials for vaccines against GBM.

NCT Number	Treatment	Summary of Results	Indication	References
NCT00643097	EGFRvIII peptide vaccine + DI-TMZ	EGFRvIII-expressing cells eradicated and vaccine immunogenic, with DI-TMZ cohort having enhanced humoral response. Median overall survival of 23.6 months.	Primary GBM	Sampson et al. [13] Sampson et al. [14]
NCT00458601	EGFRvIII peptide vaccine + TMZ	Median overall survival of 21.8 months and 36-month survival of 26%. Anti-EGFRvIII antibodies increased ≥4-fold in 85% of patients with duration of treatment.	Primary GBM	Schuster et al. [15]
NCT01480479	EGFRvIII peptide vaccine + TMZ	Strong humoral responses; however, no survival advantage and loss of EGFRvIII expression upon recurrence.	Primary GBM	Weller et al. [16]
NCT01498328	EGFRvIII peptide vaccine + bevacizumab	24-month survival of 20% compared to 3% for controls.	Recurrent GBM	Reardon et al. [17]
NCT00639639	CMV pp65 DC vaccine + Td Toxoid + TMZ	Td toxoid pre-conditioning enhanced DC migration to the lymph nodes and improved survival. 3/6 Td toxoid patients were alive and progression-free at time of survival analysis (>36.6 months), while controls had median overall survival of 18.5 months.	Primary GBM	Mitchell et al. [18]
NCT00639639	CMV pp65 DC vaccine + DI-TMZ	Antigen-specific immune responses and median overall survival of 41.1 months in DI-TMZ cohort. A total of 36% survival 5 years from diagnosis, with four patients remaining progression-free at 59–64 months from diagnosis.	Primary GBM	Batich et al. [19]
NCT02366728	CMV pp65 DC vaccine + 111In-labeled DC vaccine + Td Toxoid + basiliximab	Ongoing, have reported increased DC migration to lymph nodes following Td toxoid pre-conditioning.	Primary GBM	Batich et al. [20]
NCT02454634	IDH1 peptide vaccine	A total of 93% vaccine-specific response rate, 84% survival >3 years.	High-grade glioma	Platten et al. [21]
NCT00045968	DCVax-L vaccine	Median overall survival of 23.1 months, with large group (*n =* 100) reaching 40.5 months.	Primary GBM	Liau et al. [22]
NCT00293423	HSPPC-96 peptide vaccine	Specific immune response in 11 of the 12 patients, responders had median overall survival of 11.8 months.	Recurrent GBM	Crane et al. [23] Bloch et al. [24]
NCT02122822	HSPPC-96 peptide vaccine + TMZ + radiotherapy	Median overall survival of 31.4 months. Patients with high tumor-specific immune responses had median overall survival of >40.5 months compared to 14.6 months for low responders.	Primary GBM	Ji et al. [25]
NCT00905060	HSPPC-96 vaccine + TMZ	Median overall survival of 23.8 months. Patients with low PD-L1 expression in myeloid cells had median overall survival of 44.7 months compared to 18 months for those with high expression.	Primary GBM	Bloch et al. [26]

**Table 2:** Summary of clinical trials for immune checkpoint inhibitors against GBM.

NCT Number	Treatment	Summary of Results	Indication	References
NCT02017717	Nivolumab (anti-PD-1) or bevacizumab	Median overall survival was around 10 months for both groups; 12-month survival rates were identical between treatments at 42%.	Recurrent GBM	Reardon et al. [46]
NCT02617589	Nivolumab + radiotherapy or TMZ + radiotherapy	No survival advantage over TMZ, median overall survival of 13.4 months for nivolumab cohort and 14.88 months for TMZ.	Primary GBM	No Reference
NCT02667587	Nivolumab + TMZ + radiotherapy	Nivolumab provided no survival advantage over placebo, trial still ongoing.	Primary GBM	Squibb et al. [47]
NCT02313272	Hypofractionated stereotactic irradiation + pembrolizumab (anti-PD-1) + bevacizumab	>50% patients had significant response; median overall survival of 13.5 months.	Recurrent high-grade glioma	Sahebjam et al. [48]
NCT02337491	Pembrolizumab or pembrolizumab + bevacizumab	Median overall survival of 8.8 months for pembrolizumab with bevacizumab, 10.3 months for pembrolizumab alone.	Recurrent GBM	Reardon et al. [49]
NCT02550249	Neoadjuvant nivolumab	Neoadjuvant nivolumab enhanced chemokine expression, TCR clonal diversity among TILs and immune cell infiltration of the tumor; however, median overall survival was only 7.3 months.	GBM	Schalper et al. [50]
NCT02336165	Durvalumab (anti-PD-L1) alone, with bevacizumab or with radiotherapy	Preliminary results of recurrent, bevacizumab-refractory cohort had 36% survival at 5.5 months. Trial still ongoing.	GBM	Reardon et al. [51]
NCT02658981	Anti-LAG-3 or anti-4-1BB alone or with anti-PD-1	Median overall survival of 8 months for anti-LAG-3, 7 months for anti-LAG-3, anti-PD-1 combination and 14 months for anti-4-1BB. Trial still ongoing.	Recurrent GBM	Lim et al. [52]

**Table 3:** Summary of clinical trials for CAR T cells against GBM.

NCT Number	Treatment	Summary of Results	Indication	References
NCT00730613	IL13(E13Y)-CD3ζ CAR T cells (first generation)	Transient inflammation at tumor site and a significant decrease in IL13Rα2 expression post-treatment were observed. Two grade 3 adverse events were observed. A median survival of 11 months after tumor relapse was noted.	Recurrent GBM	Brown et al. [90]
NCT02208362	IL13(E13Y)-41BBζ CAR T cells (second generation)	A single patient with multifocal relapsed GBM was treated, resulting in 77–100% decrease in tumor burden and 7.5 months of progression-free survival. Increased presence of inflammatory cytokines at tumor site with no adverse events related to CAR T cell therapy.	Recurrent GBM	Brown et al. [91]
NCT01109095	HER2-CD28ζ CAR T cells (second generation)	No dose-limiting toxicity was observed and CAR T cells persisted for 12 months post-infusion. No significant increase in survival was noted, with a median overall survival of 11.1 months.	GBM	Ahmed et al. [98]
NCT02209376	EGFRvIII-41BBζ CAR T cells (second generation)	No dose-limiting toxicity was observed and EGFRvIII expression was reduced post-treatment. No significant increase in survival was noted, with a median overall survival of 8 months post-treatment.	Recurrent GBM	O’Rourke et al. [87]
NCT01454596	EGFRvIII-CD28-41BBζ CAR T cells (third generation)	At highest dose, 2 patients suffered dose-limiting toxicity. A median overall survival of 6.9 months was noted, with one patient alive at 59 months.	Recurrent GBM	Goff et al. [99]
